# Urinary dysfunction in myasthenia Gravis: a cross-sectional case-control study

**DOI:** 10.1007/s10072-025-08588-8

**Published:** 2026-01-03

**Authors:** Kamel Shihada, Alon Gorenshtein, Gil I. Wolfe, Shahar Shelly

**Affiliations:** 1 Department of Neurology, Rambam Medical Center, Haifa, Israel; 2 Azrieli Faculty of Medicine, Bar-Ilan University, Safed, Israel; 3 Department of Neurology, Jacobs School of Medicine and Biomedical Sciences, University at Buffalo/SUNY, Buffalo, NY USA; 4 Neuroimmunology Laboratory, Ruth, and Bruce Rappaport Faculty of Medicine, Technion – Israel Institute of Technology, Haifa, 3525408 Israel; 5 Department of Neurology, Mayo Clinic, Rochester, MN USA

**Keywords:** Urinary incontinence, Myasthenia gravis, Overactive bladder, Pyridostigmine, MG-ADL

## Abstract

**Introduction:**

Myasthenia gravis (MG) is a disorder of neuromuscular transmission that primarily affects skeletal muscles, leading to cranial, truncal, and limb weakness. While motor symptoms are well documented, the prevalence and clinical significance of urinary dysfunction particularly urinary incontinence (UI) and overactive bladder (OAB) symptoms remain underexplored in MG, with previous reports limited to small cohorts.

**Methods:**

In this study, 86 MG patients and 90 age- and sex-matched inpatient controls were evaluated. All participants completed the International Consultation on Incontinence Questionnaire-Short Form (ICIQ-UI SF) and the Overactive Bladder Symptom Score (OABSS). MG severity was assessed using the Myasthenia Gravis Activities of Daily Living (MG-ADL) scale. MG patients were further categorized into early-onset (EOMG), late-onset (LOMG), and very late-onset (VLOMG) subgroups. Logistic regression identified predictors of UI, and Kaplan–Meier analysis was used to evaluate time to first UI symptoms.

**Results:**

MG patients had significantly higher prevalence of UI symptoms (ICIQ-UI SF ≥ 6 in 52.3% vs. 12.2% in controls; *p* < 0.001). LOMG patients exhibited the highest UI prevalence (63.2%) and progressed most rapidly to UI, while EOMG patients had the latest onset. OAB symptoms were also more prominent in MG, marked by higher OABSS and increased urgency. MG-ADL score independently predicted UI (OR 2.38; 95% CI 1.04–5.46; *p* = 0.041).

**Discussion:**

UI and OAB symptoms are more common in MG patients than in controls. MG-ADL score is an independent predictor of UI, and age at disease onset may influence the timing of UI development. Early identification and intervention may improve patient outcomes.

**Supplementary Information:**

The online version contains supplementary material available at 10.1007/s10072-025-08588-8.

## Introduction

Myasthenia gravis (MG) is an immune-mediated chronic neuromuscular junction (NMJ) disorder, characterized by fatigable weakness of skeletal muscles due to autoantibody-mediated attack on the postsynaptic membrane [1]. Approximately 80% of patients exhibit elevated levels of antibodies targeting the nicotinic acetylcholine receptor (AChR), impairing synaptic transmission [2]. Muscle-specific kinase (MuSK) antibodies are present in 5%–8% of all MG patients, and interfere with the clustering of AChR at NMJs [3]. Clinically, MG presents with varied constellations of ocular symptoms (e.g., ptosis, diplopia), bulbar involvement (e.g., dysphagia, dysarthria), trunk and limb weakness and dyspnea [4]. 

Urinary incontinence (UI) is a known clinical manifestation of various neurologic diseases such as multiple sclerosis (MS), Parkinson’s disease (PD), stroke, and spina bifida, often arising due to neurogenic detrusor overactivity leading to overactive bladder (OAB) symptoms [5, 6]. In MG, urinary dysfunction has been reported, but only in small cohorts, and its significance remains uncertain. Pathophysiological mechanisms potentially linking MG to urinary dysfunction are limited and underexplored.

Micturition, or urination, is a complex process requiring precise coordination between somatic and autonomic pathways driven by a finely tuned network of excitatory and inhibitory neural signals, acting primarily on the detrusor muscle, internal urethral sphincter, and external urethral sphincter (EUS) [7]. Importantly, the EUS is composed of skeletal striated muscle under voluntary control that contracts to prevent leakage but relaxes during voiding via the deep perineal branch of the pudendal nerve which acts on nicotinic receptors [8]. Interestingly, studies have reported that pelvic floor muscles, particularly EUS, are susceptible to weakness and fatigue [9]; this is considered a primary mechanism causing stress UI [10]. Since skeletal muscle fatigability and weakness are hallmark features of MG, it is plausible that MG may be associated with urinary dysfunction.

In addition, other factors may contribute to urinary symptoms in MG, including overall comorbidity burden and functional disability [11], as well as treatment-related effects such as pyridostigmine use [12]. 

A few case reports have documented urinary dysfunction among MG patients, describing presentations ranging from overactive bladder to detrusor areflexia [13–17]. To date, only three small case-control studies have reported a significant increase in OAB symptoms in MG based on questionnaires assessments [18–20]. Although these were limited by small sample sizes and the absence of standardized cutoff criteria for defining clinically relevant UI. Akan et al. further utilized uroflowmetry and ultrasonography in 36 MG patients who were free of cholinesterase inhibitor treatment, demonstrating increased urinary dysfunction, particularly prominent among late-onset patients [20]. These preliminary findings, although informative, highlight the need for larger controlled studies that employ validated questionnaires with standardized cutoffs and incorporate disease severity measures such as the MG-ADL.

Validated standardized questionnaires are widely used to assess urinary symptoms. The International Consultation on Incontinence Questionnaire – Urinary Incontinence Short Form (ICIQ-UI SF) evaluates the presence, severity and impact of urinary incontinence on daily life based on standardized cutoffs [21]. The Overactive Bladder Symptom Score (OABSS) assesses the severity of OAB symptoms [22]. 

We investigated the association between MG and UI by administering the ICIQ-UI SF and OABSS to MG patients and inpatient age- and sex-matched controls. By comparing the prevalence and severity of urinary symptoms between these groups, we aimed for a more comprehensive understanding of urinary dysfunction in MG, extending beyond OAB symptoms alone to include other UI subtypes. Furthermore, we evaluated whether more pronounced urinary symptoms correlate with greater MG severity.

## Methods and materials

### Participants and recruitment

The study was approved by the Ethics Committee of Rambam Health Care Campus, and informed consent was obtained from all participants prior to enrollment.

MG subjects were diagnosed at our tertiary academic medical center and were receiving ongoing medical care in our neurology department. Information regarding MG diagnosis, antibody status, current medical treatment, and disease comorbidities were retrieved from electronic medical records. Clinical severity of MG is routinely assessed at all visits using the eight-item MG-ADL profile [23], and documented by a certified neurologist. Scored from 0 to 24, higher MG-ADL scores indicate more severe disease.

Age- and sex-matched controls without a diagnosis of MG were recruited. The control group consisted of patients attending neurological consultations at internal departments or emergency departments for common neurological complaints, which were determined to be non-neurological by our consultation.

### Exclusion criteria

Exclusions included patients under the age of 18 years, congenital myasthenic syndromes, cases in which the diagnosis of MG was uncertain or not confirmed by clinical, serological, or electrophysiological evidence, and severe cognitive impairment or communication barriers preventing questionnaire completion, and clinically suspected or documented urinary tract infection at the time of assessment.

Patients with central nervous system (CNS) disorders expected to substantially affect urinary function (e.g., multiple sclerosis, intracranial tumor, normal pressure hydrocephalus, active neurodegenerative disease) were excluded based on medical-record review (neurology notes, problem lists, discharge summaries). Routine brain MRI was not required, and a prior ischemic stroke was not an exclusion criterion.

### Subgroup classification

A. Age at Onset Subgroups:

MG patients were subdivided into three populations based on age at MG symptoms onset:

1. Early-Onset MG (EOMG) for symptom onset < 50 years; (2) Late-Onset MG (LOMG) for symptom onset 50 to < 65 years; and (3) Very Late-Onset MG (VLOMG) with symptom onset ≥ 65 years [24].

B. Disease Severity Subgroups:

Although the MG-ADL is widely used and well validated, no standard cutoff exists to classify severity into discrete categories. For the present analysis, we chose an MG-ADL score of ≥ 6 to represent higher disease severity, as this threshold is now routinely used as an inclusion criterion in modern phase 2–3 randomized controlled trials of gMG, including the MINT (inebilizumab) [25], RemeMG (telitacicept) [26], MaGic (DNTH103) [27].

### Assessment of urinary incontinence and urinary symptoms

To assess urinary incontinence (UI) symptoms, all subjects completed two validated surveys during inpatient evaluation or follow-up appointments. The ICIQ-UI SF consists of four questions, with a final score ranging from 0 to 21. It evaluates the presence, frequency, severity, and impact of urinary incontinence on daily life. A score of ≥ 6 is widely recognized as a strong indicator of urinary incontinence, based on established severity categories and supported by clinical research findings [28, 29]. Additionally, UI severity can be categorized as slight (1–5), moderate (6–12), severe (13–18), or very severe (19–21) [30]. The survey includes a self-diagnostic question regarding the trigger for urine leakage to help indicate the UI subtype. For example, if leakage occurs when coughing, sneezing, or during physical activity/exercise, stress incontinence is deemed present. The OABSS assesses severity and frequency of overactive bladder (OAB) symptoms [22]. It contains four questions to evaluate daytime frequency, nighttime frequency (nocturia), urgency and urge incontinence, with total scores ranging from 0 to 15, with a higher score reflecting greater severity.

### Additional prespecified analyses

In addition to the primary MG-control comparisons, we conducted four complementary analyses: (i) sex-stratified comparisons across the main urinary endpoints with Bonferroni-adjusted pairwise comparisons (Supplementary Tables S4a-S4b); (ii) summarized, within MG only, the association of multiparity (women; OR per additional birth) and BPH (men; BPH + vs. BPH−) with urinary outcomes (Supplementary S5); (iii) an older-onset analysis in LOMG and VLOMG modeling multimorbidity (≥ 2 vs. 0–1 comorbidities), with comorbidity burden was summarized as a count of recorded diagnoses and operationalized as multimorbidity (≥ 2) vs. 0–1, consistent with consensus definitions and prior analytic practice grouping burden as 0, 1, and ≥ 2 [31] (Supplementary S6), and (iv) a pyridostigmine comparison (users vs. non-users) (Supplementary S7).

### Time-to-event analysis

Kaplan-Meier survival analysis was performed to estimate the time from MG onset to the first episode of urinary incontinence (UI). This analysis included only MG patients who currently reported to have UI (defined as an ICIQ-UI SF total score ≥ 6) at the time of questionnaire completion. The event for this analysis was defined specifically as the first episode of UI symptoms, regardless of initial severity, as recalled retrospectively by each patient. Thus, for each MG patient currently with UI, the survival time was calculated as the interval (in years) from the date of MG symptom onset until the first recalled UI episode (event). We further illustrated this by analyzing the median time to first recalled UI symptoms (T50), defined as the time by which 50% of MG patients had developed urinary incontinence symptoms after the onset of their MG symptoms. Median times were derived from Kaplan-Meier curves and are presented in Supplementary Fig. 2a + 2b. Given the retrospective nature of these data, the time-to-event analysis should be regarded as exploratory and interpreted with caution due to the potential for recall bias and limited precision.

### Statistical analysis

Continuous variables were reported as means (± standard deviations) or medians (interquartile ranges). Between-group comparisons of continuous outcomes were conducted using Student’s t test, the Mann–Whitney U test, or the Kruskal–Wallis test for more than two groups if data were non-normally distributed. Categorical variables were compared using the chi-square test or Fisher’s exact test when expected cell counts were < 5. Pairwise comparisons among the three MG subgroups (EOMG, LOMG, VLOMG) employed Bonferroni adjustment for multiple comparisons. Correlation analyses were performed using Pearson’s or Spearman’s coefficient, depending on normality. Logistic regression models (univariate followed by multivariate) were constructed to identify independent predictors of UI, controlling for potential confounders. Kaplan–Meier analysis was used to evaluate time-to-event data (onset of urinary incontinence), and the log-rank test assessed differences among subgroups. *P* < 0.05 was considered statistically significant.

## Results

### Demographic and clinical characteristics of the study population

A total of 86 MG patients and 90 matched controls were enrolled (Table 1). The two groups were similar in mean age (59.6 ± 16.5 years in MG vs. 59.5 ± 16.9 years in controls, *p* = 0.974) and sex distribution (55.8% vs. 55.6% male, respectively; *p* = 0.970). Comorbidities were broadly comparable, although prior stroke was more prevalent in controls (1.1% vs. 12.2%; *p* = 0.006). Among MG patients, EOMG was the most common subtype (47.7%), followed by VLOMG (30.2%) and (LOMG, 22.1%). Most patients (77.9%) were seropositive for AChR antibodies. (Table 1). Controls were mostly healthy individuals or patients with general internal medical conditions, explicitly excluding those with a primary neurological diagnosis.

**Table 1:** Demographic and clinical characteristics of the study population

	MG patients (86)	Controls (90)	*P*-value
Age	59.6 ± 16.5	59.5 ± 16.9	0.974
Sex (Males)	48 (55.8%)	50 (55.6%)	0.970
**Comorbidities**			
DM	22 (25.6%)	22 (24.4%)	0.859
HF	2 (2.3%)	3 (3.3%)	0.697
CKD	3 (3.4%)	4 (4.4%)	0.752
History of stroke	1 (1.1%)	11 (12.2%)	**0.006**
Pelvic organs prolapse	1 (1.1%)	0 (0.0%)	0.302
BPH	14 (16.2%)	8 (8.9%)	0.127
Pelvic surgery	12 (14.0%)	11 (12.2%)	0.731
Spinal surgery	2 (2.3%)	3 (3.3%)	0.685
Smoking	8(9.3%)	8 (8.9%)	0.884
Multiparous (Para 2 or more)	23(26.74%)	25 (27.77%)	0.857
Anxiety	3(3.5%)	2(2.2%)	0.608
Depression	4 (4.7%)	1(1.1%)	0.157
**MG characteristics**			
MG age of onset (yrs)	49.4	-	-
EOMG	41(47.7%)	-	-
LOMG	19(22.1%)	-	-
VLOMG	26 (30.2%)	-	-
MG duration (yrs)	10.18		
**Serology**			
Ach (+)	67 (77.9%)	-	-
Ach (-)	8 (9.3%)	-	-
Anti MuSK (+)	5 (5.8%)	-	-
MG clinical severity			
MG-ADL	4.5 ± 3.9	-	-
**MG at presentation**			
Pure Ocular	33 (38.4%)	-	-
Pure Bulbar	0 (0.0%)	-	-
Limb muscle weakness	3 (3.5%)	-	-
Respiratory muscle weakness	0 (0.0%)	-	-
Thymic mass + oculobulbar weakness leads to diagnosis	11 (12.8%)	-	-
Ocular + Bulbar	4 (4.7%)	-	-
Ocular + Limb muscle weakness	3 (3.5%)	-	-
Bulbar + Limb muscle weakness	1 (1.2%)	-	-
Ocular + Bulbar + Limb muscle weakness	6 (7%)	-	-
Ocular + Respiratory muscle weakness	20 (23.3%)	-	-
Not specified**	5 (5.8%)	-	-
**MG treatment**			
Pyridostigmine only	9 (10.5%)	-	-
Pyridostigmine + immunotherapies*	35 (40.7%)		
Immunotherapies alone	39 (45.3%)	-	-
No treatment	3 (3.5%)	-	-

*immunotherapies include: Mycophenolate mofetil, Azathioprine Rituximab, Efgartigimod alfa, IVIG, Prednisone, Plasma exchange, Methotrexate not specified* data were unavailable or unrecorded in the patient medical records. *Abbreviations*: *MG* myasthenia gravis, *EOMG* early-onset MG, *LOMG* late-onset MG, *VLOMG* very late-onset MG, *AChR* acetylcholine receptor, *MuSK* muscle-specific kinase, *MG-ADL* Myasthenia Gravis Activities of Daily Living, *DM* diabetes mellitus, *HF* heart failure, *CKD* chronic kidney disease, *BPH* benign prostatic hyperplasia

### Urinary symptoms

An ICIQ-UI SF score ≥ 6, is the cutoff highest suggestive of UI, was significantly more frequent in MG patients than in controls (52.3% vs. 12.2%, *p* < 0.001; Table 2). All MG subtypes had higher rates of UI relative to controls, with LOMG showing the highest prevalence (63.2%) (supplementary Fig. 1a). Additionally, about one-third of MG patients reported severe incontinence (ICIQ-UI SF score: 13–18), compared to only 2.2% of controls. Stress incontinence symptoms, as assessed by the ICIQ-UI SF self-diagnostic question, was also markedly higher in MG patients compared to controls (31.4% vs. 6.7%, *p* < 0.001). OABSS was also more pronounced in MG (*p* < 0.001) indicating more severe symptoms (supplementary Fig. 1b). While increased daytime frequency (≥ 8 times/day) was not statistically significant (*p* = 0.331), nighttime frequency (≥ 3 times/night) was more common among MG patients (27.9% vs. 11.1%, *p* = 0.004). Urgency episodes occurring ≥ 2 times/day, a common OAB symptom, were strikingly more common in MG, with 38.4% of patients reporting such episodes compared to only 2.2% of controls (*p* < 0.001) (Table 2).

**Table 2:** Urinary symptoms analysis in MG patients and controls

Variable	Total MG (*n* = 86)	EOMG (*n* = 41)	LOMG (*n* = 19)	VLOMG (*n* = 26)	Controls (*n* = 90)	*P*-value*
Urinary incontinence (ICIQ UI-SF score ≥ 6), n (%)	45 (52.3)	21 (51.2) †	12 (63.2) †	12 (46.2) †	11 (12.2)	**< 0.001**
**ICIQ UI-SF severity classification**,** n (%)**						
Slight (1–5)	4 (4.7)	2 (4.9)	2 (10.5)	0 (0.0)	2 (2.2)	0.358
Moderate (6–12)	13 (15.1)	5 (12.2)	5 (26.3)	3 (11.5)	9 (10.0)	0.292
Severe (13–18)	28 (32.6) †	14 (34.1) †	6 (31.6) †	8 (30.8) †	2 (2.2)	**< 0.001**
Very severe (19–21)	4 (4.7)	2 (4.9)	1 (5.3)	1 (3.8)	0 (0.0)	0.039
Stress urinary incontinence, n (%)	27 (31.4) †	13 (31.7) †	9 (47.4) †	5 (19.2)	6 (6.7)	**< 0.001**
Daytime frequency ≥ 8 times, n (%)	17 (19.8)	8 (19.5)	5 (26.3)	4 (15.4)	13 (14.4)	0.331
**Nighttime frequency**,** n (%)**						
1 time	21 (24.4)	9 (22.0)	4 (21.1)	8 (30.8)	36 (40.0)	0.025
2 times	22 (25.6)	11 (26.8)	4 (21.1)	7 (26.9)	13 (14.4)	0.054
≥ 3 times	24 (27.9) †	9 (22.0)	8 (42.1) †	7 (26.9)	10 (11.1)	**0.004**
Any nighttime void (≥ 1 episode/night)	67 (77.9)	29 (70.7)	16 (84.2)	22 (84.6)	59 (65.5)	0.152
OABSS score [IQR]	6.0 [1.0–10.0] †	6.0 [1.0–10.0]	7.0 [3.0–10.0] †	3.5 [1.0–11.0] †	1.0 [0.0–3.0]	**< 0.001**
**Urgency**,** n (%)**						
Once a week or more	3 (3.5)	1 (2.4)	1 (5.3)	1 (3.8)	2 (2.2)	0.611
About once a day	10 (11.6)	8 (19.5) †‡	2 (10.5)	0 (0.0)	5 (5.6)	0.129
2–4 times a day	17 (19.8) †	5 (12.2) †	4 (21.1) †	8 (30.8) †	1 (1.1)	**< 0.001**
5 times a day or more	16 (18.6) †	8 (19.5) †	5 (26.3) †	3 (11.5) †	1 (1.1)	**< 0.001**
Duration of urinary symptoms (years) [IQR]	0.4 [0.0–1.0] †	0.2 [0.0–1.0] †‡	1.0 [0.0–5.0]	0.2 [0.0–1.0] †	2.0 [1.5–11.0]	**< 0.001**

*Abbreviations*: *EOMG* early-onset myasthenia gravis, *LOMG* late-onset myasthenia gravis, *VLOMG* very late-onset myasthenia gravis, *ICIQ-UI SF* International Consultation on Incontinence Questionnaire-Urinary Incontinence Short Form, *OABSS* Overactive Bladder Symptom Score, *IQR* interquartile range

*P*-values compare the four groups (EOMG, LOMG, VLOMG, and controls) using χ² test unless otherwise indicated. † Significantly different from control group after Bonferroni correction (*p* < 0.0167). ‡Significant difference between MG subgroups after Bonferroni correction (*p* < 0.0167). Total MG column provided for descriptive purposes only; not included in statistical comparisons. Bonferroni correction was applied for multiple comparisons with adjusted significance threshold *p* < 0.0167 for primary comparisons between each MG subgroup and controls. See supplementary table S1 for detailed pairwise comparisons

Pairwise comparisons among MG subgroups and controls revealed that all three MG subgroups (EOMG, LOMG, and VLOMG) had significantly higher rates of overall urinary incontinence (*p* < 0.001) and more severe ICIQ-UI SF scores (*p* < 0.001). They also exhibited more pronounced OAB symptoms indicated by higher OABSS scores and increased urgency episodes. Post hoc comparisons among the three MG subgroups themselves did not reach statistical significance after Bonferroni correction (supplementary Tables).

### Sex-Stratified analyses

In sex-stratified analyses, MG was associated with a higher burden of urinary symptoms in both men and women (Supplementary Tables S4a-S4b). Among men, urinary incontinence (ICIQ-UI SF ≥ 6) was present in 43.8% of MG patients versus 14.0% of male controls (Bonferroni-adjusted *p* = 0.009), and among women in 63.2% of MG patients versus 10.0% of female controls (*p* < 0.001). Total ICIQ-UI SF and OABSS scores were higher in MG than in controls within each sex. Stress urinary incontinence and urgency were particularly frequent in female MG patients (stress UI 55.3% vs. 12.5% and urgency 60.5% vs. 2.5% in female controls; both *p* < 0.001), although urgency was also more common in male MG patients compared with male controls (41.7% vs. 12.0%; *p* = 0.007).

### Correlation analysis

Correlation coefficients between urinary symptom measures and MG-disease are summarized in Table 3. Total ICIQ-UI SF score correlated moderately with both OABSS (*r* = 0.863, *p* = 0.001) and voiding frequency (daytime and nighttime; both *p* = 0.001). Importantly, MG-ADL scores, indicative of disease severity, exhibited a modest association with both the ICIQ-UI SF score (*r* = 0.287; *p* = 0.010) and the OABSS (*r* = 0.228; *p* = 0.050).

**Table 3:** Correlation analysis of urinary symptoms in MG patients

Clinical Variable	Total ICIQ UI-SF Score		OABSS Final Score	
	r	*p*-value	r	*p*-value
Disease Severity Measures				
MG-ADL Score	0.287	**0.010**	0.228	**0.050**
Patient Characteristics				
Age	0.034	0.200	0.159	0.200
Disease Duration (years)	0.187	0.100	0.187	0.100
Age at Onset	−0.065	0.200	0.041	0.200
Urinary Symptom Measures				
Total ICIQ UI-SF Score	1.000	-	0.863	**0.001**
OABSS Final Score	0.863	**0.001**	1.000	-
Duration of Urinary Symptoms (years)†	0.398	**0.001**	0.290	**0.010**
Daytime Frequency (number of voids)	0.354	**0.001**	0.525	**0.001**
Nighttime Frequency (number of voids)	0.457	**0.001**	0.655	**0.001**

*Abbreviations*: *ICIQ-UI SF* International Consultation on Incontinence Questionnaire-Urinary Incontinence Short Form, *OABSS* Overactive Bladder Symptom Score, *MG-ADL* Myasthenia Gravis Activities of Daily Living

### Predictors of urinary incontinence

**Fig. 1:**
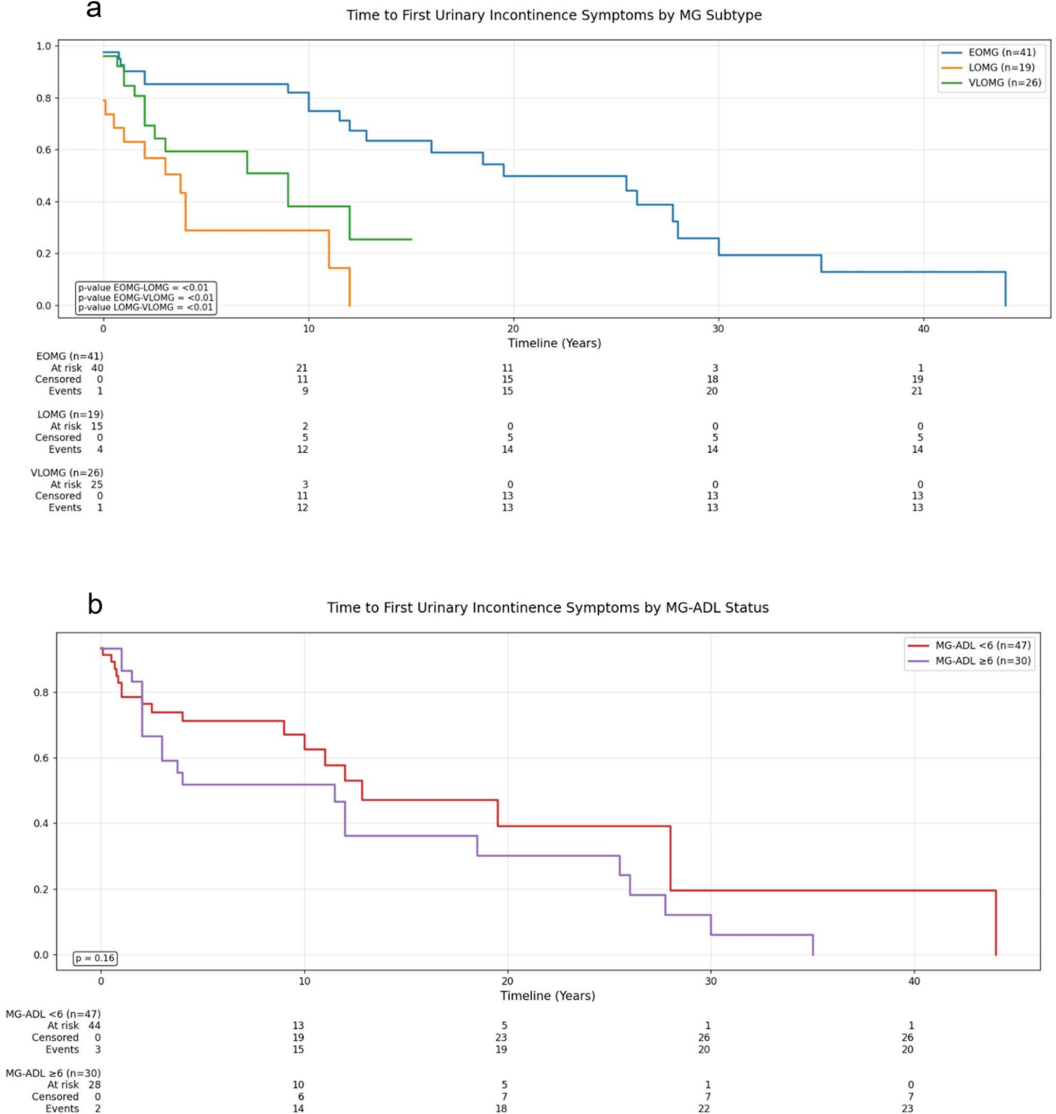
Kaplan-Meier Analysis of Time to Urinary Incontinence Among Myasthenia Gravis Patients: **a**. Kaplan-Meier illustrates time to first urinary incontinence symptoms among patients with early-onset (EOMG), late-onset (LOMG), and very late-onset (VLOMG) myasthenia gravis, LOMG progressed to incontinence more rapidly than EOMG and VLOMG (all *p* < 0.01). **b**. Kaplan-Meier estimates of time to first urinary incontinence symptoms among patients with myasthenia gravis, stratified by disease severity (MG-ADL < 6vs. ≥6), patients with higher MG-ADL scores tended to reach incontinence sooner, but the difference was not statistically significant (*p* = 0.16). Abbreviations: ICIQ UI-SF, International Consultation on Incontinence Questionnaire-Urinary Incontinence Short Form; MG-ADL, Myasthenia Gravis Activities of Daily Living

On univariate analysis (Table 4), higher MG-ADL scores were significantly associated with increased odds of meeting the ICIQ-UI SF threshold for urinary incontinence (OR 2.57, *p* = 0.050). This association was further reinforced and confirmed by multivariate logistic regression (supplementary table S2), which identified MG-ADL as a statistically significant independent risk factor of UI (adjusted OR 2.38, 95% CI 1.04–5.46; *p* = 0.041). Neither pyridostigmine use, disease duration, nor diuretic medications achieved significance in either model, indicating that overall disease severity, as assessed by MG-ADL, was the primary driver of increased UI risk among MG patients. Among MG patients with clinically relevant urinary incontinence (ICIQ-UI SF ≥ 6), time from MG symptom onset to UI development varied significantly by MG subtype (Fig. 1a; overall *p* < 0.01). The LOMG subgroup showed the fastest progression, with a median onset time of 3.8 years, significantly earlier than VLOMG (median 9.0 years, *p* < 0.01) and EOMG, which had the slowest progression (median 19.5 years, *p* < 0.01) (supplementary Fig. 2a). When stratifying by disease severity (Fig. 1b), patients with MG-ADL ≥ 6 developed UI somewhat earlier than those with MG-ADL < 6 with median time 11.5 vs. 12.8 years, respectively; (supplementary Fig. 2b), however, this difference did not reach statistical significance (*p* = 0.16). These findings suggest that the age of MG onset has a greater influence on time to UI than disease severity, though it should be interpreted with caution given reliance on retrospective recall.

**Table 4:** Univariate analysis for predicting urinary incontinence (ICIQ UI-SF ≥ 6)

Variable Category	Variable	Odds Ratio	95% CI	*p*-value
Demographics				
	Age	1.02	0.99–1.05	0.174
	Male Gender	0.85	0.36–2.03	0.725
Disease Characteristics				
	MG-ADL Score	2.57	1.13–5.83	**0.050**
	EOMG	0.92	0.39–2.14	0.200
	LOMG	1.77	0.62–5.04	0.200
	VLOMG	0.71	0.28–1.81	0.469
	AchR Positive	1.32	0.47–3.71	0.598
	MuSK Positive	1.45	0.22–9.52	0.701
	Disease Duration	1.10	0.98–1.24	0.126
Treatment				
	Pyridostigmine Only	0.23	0.04–1.16	0.100
	Pyridostigmine + immunotherapies	1.18	0.48–2.89	0.200
	Any Pyridostigmine Use	1.00	0.43–2.32	0.200
	Immunotherapies alone	1.30	0.55–3.08	0.200
Comorbidities				
	Diabetes Mellitus	1.27	0.47–3.47	0.639
	Heart Failure	0.98	0.11–8.92	0.967
	Chronic Kidney Disease	0.88	0.12–6.49	0.896
	History of Stroke	0.92	0.05–15.80	0.912
	Smoking	1.05	0.32–3.46	0.952
	Pelvic Surgery	0.87	0.25–3.05	0.841
	Spinal Surgery	0.72	0.09–5.71	0.821
	Anxiety	2.65	0.25–28.23	0.417
	Depression	3.89	0.38–39.52	0.251
Medications				
	Diuretics	2.13	0.61–7.41	0.235
	Alpha Blockers	1.92	0.33–11.19	0.471
	Anticholinergics	0.42	0.04–4.71	0.484
	Narcotics	1.32	0.11–15.22	0.882
	Sedatives	0.92	0.29–2.94	0.892
	Antihistamines	1.48	0.23–9.52	0.682
	ACE-Inhibitors/ARBs	1.17	0.37–3.70	0.786

*Abbreviations*: *ICIQ-UI SF* International Consultation on Incontinence Questionnaire-Urinary Incontinence Short Form, *OABSS* Overactive Bladder Symptom Score, *MG-ADL* Myasthenia Gravis Activities of Daily Living

A supplementary table summarizing medication use among MG patients is provided (Supplementary Table S3). The wide confidence intervals observed for some medications (e.g., narcotics, alpha blockers) likely reflect the small number of patients receiving these treatments

### Obstetric/prostatic factors, comorbidities, and pyridostigmine

Because obstetric and prostatic factors may influence lower urinary tract symptoms, we examined multiparity in women and benign prostatic hyperplasia (BPH) in men within the MG cohort. Neither multiparity nor BPH was significantly associated with urinary incontinence, stress UI, or nocturia; odds ratios were close to 1.0 with 95% confidence intervals including unity (Supplementary Table S5). Among older-onset MG (LOMG + VLOMG), the prevalence of urinary incontinence did not differ significantly between patients with ≥ 2 comorbidities and those with 0–1 comorbidities (56.4% vs. 33.3%; *p* = 0.538), although the subgroup with multimorbidity was small (Supplementary Table S6). Consistent with our regression models, the prevalence of urinary incontinence was nearly identical in pyridostigmine users and non-users (52.3% vs. 52.4%; *p* = 1.00), supporting the lack of an independent association between pyridostigmine use and UI in this cohort (Supplementary Table S7).

## Discussion

In this study, we investigated the prevalence and severity of urinary incontinence in patients with MG compared with matched controls and further examined how different MG subtypes and disease severity influence urinary symptoms. Our main findings showed that MG patients, particularly those with LOMG, are more likely to experience UI, including both stress incontinence and overactive bladder symptoms (supplementary Figs. 1a + 1b). This is consistent with previous studies that have reported similar findings [18–20, 32]. Except for one case report, we are the first to show that MG patients tend to exhibit more stress UI symptoms [32]. This finding aligns with previously proposed theory that MG-related muscle weakness and fatigue may extend to the pelvic floor muscles, specifically the EUS [9], thereby impairing their ability to support normal bladder function, eventually leading to stress UI [10], although this mechanism remains speculative and warrants confirmation through objective assessments.

In sex-stratified analyses, MG was associated with higher UI and OAB burden in both men and women, with marked excess stress UI and urgency among women (Supplementary Tables S4a-S4b). Within MG, neither multiparity nor BPH was significantly related to UI, stress UI, or nocturia, suggesting that MG-specific mechanisms likely underlie much of the urinary dysfunction (Supplementary Table S5).

Additionally, we found that the MG-ADL severity score was significantly associated with both UI and OAB severity. To the best of our knowledge, no prior studies have explored the impact of MG disease severity with urinary dysfunction based on the validated and widely used MG-ADL, a patient reported outcome. Donskov et al. assessed correlations between only OAB symptoms and several MG severity scales, reporting significant associations only with QMG, MG composite, while MG-ADL findings were mentioned but not further ellaborated [18]. After adjusting for potential confounders, our analysis identified the MG-ADL score as a significant independent predictor of UI development, highlighting that MG clinical severity is a critical determinant of urinary dysfunction risk among MG patients. MG-ADL’s greater clinical utility in this context in comparison with QMG and MG-QOL15 may be attributed to its ability to capture the functional impact of symptoms as a patient-reported outcome measure [33], in line with the ICIQ-UI SF and OABSS. This shared focus on patient experience likely makes it more sensitive to detecting associations with urinary complaints compared to objective neuromuscular assessments such as the QMG or a quality-of-life instrument.

Moreover, while the increased likelihood of UI in LOMG was shown in earlier analyses and previous studies [20], our time-to-event analysis specifically revealed a more rapid progression to UI in this subgroup. In contrast, EOMG was associated with a delayed onset. We also observed a trend toward earlier development of UI in patients with more severe MG-ADL scores, consistent with the possibility that greater functional disability may contributes to urinary symptoms [11]. In LOMG/VLOMG, UI prevalence was similar in patients with ≥ 2 comorbidities versus those with 0–1 (Supplementary Table S6). Thus, comorbidity burden alone does not appear to account for the high UI rates in older-onset MG.

Cholinesterase inhibitors, such as pyridostigmine, are essential in the management of MG by increasing acetylcholine availability at NMJs [34], however they also elevate neurotransmitter levels in smooth muscle synapses including the bladder [35]. This enhanced cholinergic activity can stimulate the bladder’s detrusor muscle, potentially leading to OAB symptoms [12]. One study reported that pyridostigmine doses over 300 mg daily, and particularly over 600 mg daily, were associated with high OAB scores [18]. We did not find pyridostigmine to have a significant independent effect on the risk of developing UI. Variability in dosing regimens, treatment durations, concomitant medications, as well as potential confounding factors such as disease severity and comorbidities, could have diluted any observable effect of cholinesterase inhibitors. Interestingly, pyridostigmine has been reported to successfully treat urinary dysfunction related to an underactive detrusor by enhancing detrusor contractility [36]. Given that many MG patients experience underactive bladder symptoms [14–17], pyridostigmine might theoretically improve their urinary function rather than exacerbate incontinence. Therefore, the association between pyridostigmine and urinary symptoms in MG patients may depend on the specific subtype of urinary dysfunction present.

An important consideration is the higher prevalence of prior stroke in the control group, a known risk factor for lower urinary tract symptoms, particularly nocturia [5]. While we observed trends toward increased nighttime frequency in some MG subgroups- most notably LOMG and VLOMG - pairwise comparisons for ≥ 1 void per night did not reach statistical significance. To assess whether stroke history may have confounded our findings, we included it in our univariate regression analysis for predictors of urinary incontinence, which revealed no significant association. This suggests that stroke-related urinary dysfunction was unlikely to confound the observed association between MG and clinically relevant urinary incontinence.

Managing urinary symptoms in MG presents distinct clinical challenges. Anticholinergic medications like oxybutynin, commonly used for overactive bladder, may worsen neuromuscular transmission and are generally contraindicated in MG [37]. Although beta-3 adrenergic agonists (e.g., mirabegron) appears to be a safer alternative, their use in MG remains insufficiently studied [38]. Additionally, non-pharmacologic approaches such as pelvic floor therapy may be limited by MG-related muscle fatigue [9]. These factors emphasize the need for appropriate personalized management for urinary symptoms in MG.

Our study has some limitations, including the absence of urodynamics testing, which limited physiologic characterization and the mechanistic specificity of urinary dysfunction (e.g., distinguishing detrusor overactivity from outlet obstruction or underactive detrusor) and prevented instrument-based confirmation of symptom scores; hence, it could more precisely characterize urinary features and confirm our findings. Additionally, while we adjusted for several confounding factors, we lacked detailed data on pyridostigmine dosing, timing relative to assessment, and treatment duration, as well as complete information on body mass index, which may influence urinary symptoms. Moreover, concomitant CNS abnormalities (other than stroke) were excluded based on medical-record review; systematic brain MRI was not performed. Finally, time- to-event analysis relied on retrospectively recalled timing of first UI symptoms, which may be subject to recall bias and affect precision in estimating median onset.

Despite these limitations, this study presents several novel contributions. It is the first case-controlled analysis to simultaneously assess both stress and urgency urinary incontinence in MG using validated questionnaires, while accounting for disease subtypes and severity. The relatively large sample size and the application of standardized cutoff criteria for clinically significant UI further strengthen the validity of our findings. The use of MG-ADL as a predictive marker, alongside a time-to-event analysis, provides unique insights into the timeline and risk factors for urinary dysfunction in MG.

In conclusion, our cross-sectional, case-controlled study reveals a significant association between MG and UI, with notable differences in UI onset among MG subtypes and a clear correlation with MG disease severity. This research may offer insights into the neuromuscular mechanisms underlying urinary control, emphasizing the broader clinical implications of MG beyond its classically recognized muscles involvement. Our findings highlight the importance of inquiring about urinary symptoms in routine clinical evaluation of MG patients to enhance patient care and quality of life. Future research could focus on evaluation of UI in MG by urodynamic studies and the value of targeted interventions to mitigate its impact.

## Supplementary Information

Below is the link to the electronic supplementary material.

Supplementary Material 1

## Data Availability

The data underlying this article will be shared on reasonable request to the corresponding author.

## Declarations

KS- No disclosures

AG- No disclosures

GW- No disclosures

SS- No disclosures
