# Supplementary material for: Urinary dysfunction in myasthenia Gravis: a cross-sectional case-control study
Source: Neurol Sci. 2026 Jan 3;47(1):91. doi: 10.1007/s10072-025-08588-8 (PMC12764552; doi:10.1007/s10072-025-08588-8)
Supplement: Supplementary file 1 — Supplementary Material 1 [file 10072_2025_8588_MOESM1_ESM.docx]

**Supplementary data:**

## **Supplementary Table S1: Pairwise Comparisons Between MG Subgroups and Controls**

|  | EOMG vs Control | LOMG vs Control | VLOMG vs Control | EOMG vs LOMG | EOMG vs VLOMG | LOMG vs VLOMG |
| --- | --- | --- | --- | --- | --- | --- |
|  | p-value | p-value | p-value | p-value | p-value | p-value |
| **Urinary Symptoms** |  |  |  |  |  |  |
| Urinary incontinence (ICIQ UI-SF score≥6) | **<0.001** | **<0.001** | **<0.001** | 0.387 | 0.686 | 0.252 |
| **Total ICIQ UI-SF SCORE** |  |  |  |  |  |  |
| Slight (1-5) | 0.385 | 0.070 | 0.431 | 0.360 | 0.246 | 0.071 |
| Moderate (6-12) | 0.703 | 0.055 | 0.815 | 0.154 | 0.921 | 0.191 |
| Severe (13-18) | **<0.001** | **<0.001** | **<0.001** | 0.839 | 0.764 | 0.950 |
| Very severe (19-21) | 0.025 | 0.027 | 0.064 | 0.943 | 0.824 | 0.784 |
| Stress urinary incontinence | **<0.001** | **<0.001** | 0.054 | 0.242 | 0.262 | 0.044 |
| Daytime frequency ≥8 times | 0.574 | 0.261 | 0.983 | 0.552 | 0.668 | 0.365 |
| **Nighttime Frequency** |  |  |  |  |  |  |
| 1 time | 0.044 | 0.119 | 0.393 | 0.937 | 0.419 | 0.467 |
| 2 times | 0.089 | 0.471 | 0.138 | 0.631 | 0.993 | 0.651 |
| ≥3 times | 0.102 | **<0.001** | 0.045 | 0.107 | 0.642 | 0.286 |
| OABSS score† | 0.029 | **<0.001** | **0.011** | 0.155 | 0.252 | 0.597 |
| Any nighttime void (≥1 episode/night) | 0.559 | **0.111** | 0.062 | 0.262 | 0.194 | 0.970 |
| **Urgency** |  |  |  |  |  |  |
| Once a week or more | 0.939 | 0.462 | 0.646 | 0.571 | 0.742 | 0.820 |
| About once a day | **0.013** | 0.422 | 0.219 | 0.385 | **0.016** | 0.091 |
| 2–4 times a day | **0.005** | **<0.001** | **<0.001** | 0.371 | 0.061 | 0.467 |
| 5 times a day or more | **<0.001** | **<0.001** | **0.010** | 0.552 | 0.391 | 0.200 |
| Duration of urinary symptoms | **0.011** | 0.194 | **0.015** | **0.004** | 0.070 | 0.370 |

*Significant at p<0.05; values in bold remain significant after Bonferroni correction (p<0.0167 for MG subgroup vs control comparisons, p<0.0167 for between MG subgroup comparisons). †Mann-Whitney U test used for continuous variables; all other comparisons used χ² test or Fisher's exact test as appropriate.

**Abbreviations:** EOMG, early-onset myasthenia gravis. LOMG, late-onset myasthenia gravis. VLOMG, very late-onset myasthenia gravis.; ICIQ-UI SF, International Consultation on Incontinence Questionnaire-Urinary Incontinence Short Form; OABSS, Overactive Bladder Symptom Score.

**Supplementary table S2: Multivariate Analysis for Predicting Urinary Incontinence (ICIQ UI-SF≥6)**

| Variable | Adjusted Odds Ratio | 95% CI | P-value |
| --- | --- | --- | --- |
| MG-ADL Score | 2.38 | 1.04-5.46 | **0.041** |
| Pyridostigmine Only | 0.29 | 0.05-1.58 | 0.153 |
| LOMG | 1.61 | 0.54-4.82 | 0.395 |
| Disease Duration | 1.07 | 0.95-1.21 | 0.274 |
| Diuretics | 1.89 | 0.52-6.86 | 0.332 |

**Note:** Variables with p < 0.25 in univariate analysis were included in the multivariate model.

**Abbreviations:** MG-ADL, Myasthenia Gravis Activities of Daily Living; LOMG, late-onset myasthenia gravis.

**Supplementary table S3: Frequency of Medication Use Among MG Patients Included in the Univariate Analysis:**

| Medication Category | Number of Patients Receiving Treatment (n, %) |
| --- | --- |
| Diuretics | 7 (0.08) |
| Alpha Blockers | 9 (0.10) |
| Anticholinergics | 0 |
| Narcotics | 0 |
| Sedatives | 0 |
| Antihistamines | 1 (0.01) |
| ACE-Inhibitors/ARBs | 11(0.12) |

**Abbreviations:** ACE-Inhibitors, angiotensin-converting enzyme inhibitors; ARBs, angiotensin II receptor blockers.

**Supplementary table S4a: Urinary symptoms and scores in MG patients and controls, stratified by sex**

| Variable | MG males (n=48) | Control males (n=50) | MG females (n=38) | Control females (n=40) | P-value |
| --- | --- | --- | --- | --- | --- |
| Urinary incontinence (ICIQ UI-SF score≥6) | 21 (43.8%) | 7 (14.0%) | 24 (63.2%) | 4 (10.0%) | **<0.001** |
| Total ICIQ UI-SF SCORE | 5.7±6.7 | 1.3±3.3 | 9.5±7.6 | 1.3±3.7 | **<0.001** |
| Slight (1-5) | 1 (2.1%) | 0 (0.0%) | 3 (7.9%) | 2 (5.0%) | 0.199 |
| Moderate (6-12) | 7 (14.6%) | 7 (14.0%) | 6 (15.8%) | 2 (5.0%) | 0.436 |
| Severe (13-18) | 14 (29.2%) | 0 (0.0%) | 14 (36.8%) | 2 (5.0%) | **<0.001** |
| Very severe (19-21) | 0 (0.0%) | 0 (0.0%) | 4 (10.5%) | 0 (0.0%) | **0.002** |
| Stress urinary incontinence | 6 (12.5%) | 1 (2.0%) | 21 (55.3%) | 5 (12.5%) | **<0.001** |
| OABSS total score | 5.0±4.4 | 2.1±2.0 | 6.4±4.9 | 1.5±1.8 | **<0.001** |
| Nocturia (≥2/night) | 27 (56.2%) | 15 (30.0%) | 19 (50.0%) | 8 (20.0%) | **0.001** |
| Daytime frequency (≥8/day) | 3 (6.2%) | 0 (0.0%) | 3 (7.9%) | 1 (2.5%) | 0.214 |
| Urgency (≥ once/day) | 20 (41.7%) | 6 (12.0%) | 23 (60.5%) | 1 (2.5%) | **<0.001** |

**Footnotes:** Values are shown as n (%) for categorical variables and mean±SD for continuous variables. P-values are for overall comparison across the four groups.

**Abbreviations:** MG, myasthenia gravis; UI, urinary incontinence; ICIQ-UI SF, International Consultation on Incontinence Questionnaire-Urinary Incontinence Short Form; OABSS, Overactive Bladder Symptom Score.

**Supplementary table S4b: Pairwise comparisons between groups (Bonferroni-adjusted p-values)**

| Variable | MG Male vs Control Male | MG Male vs MG Female | MG Male vs Control Female | Control Male vs MG Female | Control Male vs Control Female | MG Female vs Control Female |
| --- | --- | --- | --- | --- | --- | --- |
| Urinary incontinence (ICIQ UI-SF≥6) | **0.009** | 0.514 | **0.004** | **<0.001** | 1.000 | **<0.001** |
| Total ICIQ UI-SF SCORE | **0.001** | 0.056 | **0.006** | **<0.001** | 1.000 | **<0.001** |
| Stress urinary incontinence | 0.341 | **<0.001** | 1.000 | **<0.001** | 0.507 | **<0.001** |
| OABSS total score | **0.012** | 1.000 | **<0.001** | **<0.001** | 0.590 | **<0.001** |
| Nocturia (≥2/night) | 0.083 | 1.000 | **0.005** | 0.464 | 1.000 | 0.050 |
| Daytime frequency (≥8/day) | 0.682 | 1.000 | 1.000 | 0.461 | 1.000 | 1.000 |
| Urgency (≥ once/day) | **0.007** | 0.768 | **<0.001** | **<0.001** | 0.760 | **<0.001** |

P-values are adjusted for multiple testing using the Bonferroni method. Comparisons are between MG and control groups within and across sexes for each variable.

**Abbreviations:** MG, myasthenia gravis; UI, urinary incontinence; ICIQ-UI SF, International Consultation on Incontinence Questionnaire-Urinary Incontinence Short Form; OABSS, Overactive Bladder Symptom Score.

**Supplementary Table S5. Association of multiparity and BPH in MG with urinary outcomes**

| Sex | Predictor | Outcome | Overall prevalence n/N (%) | Odds ratio (95% CI) | P-value |
| --- | --- | --- | --- | --- | --- |
| Female | Multiparity | UI (ICIQ-UI SF ≥6) | 24/38 (63.2%) | 1.19 (0.77-1.84) | 0.443 |
| Female | Multiparity | Stress UI | 21/38 (55.3%) | 1.11 (0.70-1.76) | 0.651 |
| Male | BPH | UI (ICIQ-UI SF ≥6) | 21/48 (43.8%) | 0.95 (0.27-3.34) | 0.936 |
| Male | BPH | Nocturia (≥2/night) | 27/48 (56.2%) | 1.05 (0.30-3.70) | 0.936 |

Supplementary table summarizing the association of multiparity (females) and benign prostatic hyperplasia (BPH, males) among MG with selected urinary outcomes. Values are shown as overall prevalence in the sex-specific subgroup and odds ratios (ORs) with 95% confidence intervals (CIs)

**Abbreviations:** BPH: benign prostatic hyperplasia; UI: urinary incontinence; ICIQ-UI SF: International Consultation on Incontinence Questionnaire-Urinary Incontinence Short Form.

**Supplementary Table S6. UI by comorbidity count for older-onset MG (LOMG+VLOMG)**

| Variable | 0-1 comorbidities (UI n/N, %) | ≥2 comorbidities (UI n/N, %) | p-value |
| --- | --- | --- | --- |
| Urinary incontinence (ICIQ UI-SF score≥6) | 22/39 (56.41%) | 2/6 (33.33%) | **0.5384** |

**Footnotes:** Multimorbidity was defined as the presence of ≥2 documented comorbid conditions (vs 0-1), based on recorded diagnoses in the medical record. P-value is for comparison of urinary incontinence prevalence between patients with 0-1 comorbidities and those with ≥2 comorbidities.

**Abbreviations:** UI, urinary incontinence (ICIQ-UI SF ≥6); ICIQ-UI SF, International Consultation on Incontinence Questionnaire-Urinary Incontinence Short Form; MG, myasthenia gravis; LOMG, late-onset myasthenia gravis; VLOMG, very late-onset myasthenia gravis.

**Supplementary Table S7. Pyridostigmine (Users vs non-users):**

| Variable | Pyridostigmine Users (UI n/N, %) | Non-users (UI n/N, %) | p-value |
| --- | --- | --- | --- |
| Urinary incontinence (ICIQ UI-SF score≥6) | 23/44 (52.27%) | 22/42 (52.38%) | 1.000 |

**Footnotes:** Comparison of urinary incontinence prevalence between pyridostigmine users and non-users in MG patients.

**Abbreviations:** UI (Urinary incontinence) = ICIQ-UI SF ≥6; MG, myasthenia gravis.

**Supplementary figure 1a: Prevalence of Urinary Incontinence in MG Patients Compared to Controls**

Supplementary figure 1a: MG patients showed a higher overall prevalence of UI compared to age- and sex-matched controls.

**Abbreviations:** MG, Myasthenia Gravis; UI, urinary incontinence.

**Supplementary figure 1b: Severity of Overactive Bladder Symptoms in MG Patients Compared to Controls**

Supplementary figure 1b: MG patients reported higher rates of OAB symptoms compared to controls, based on OABSS components.

**Abbreviations:** MG, Myasthenia Gravis; OABSS, Overactive Bladder Symptom Score.

**Supplementary figure 2a: Median Time (T50) From Onset of MG to First Urinary Incontinence (UI) Symptoms by MG Subtype**
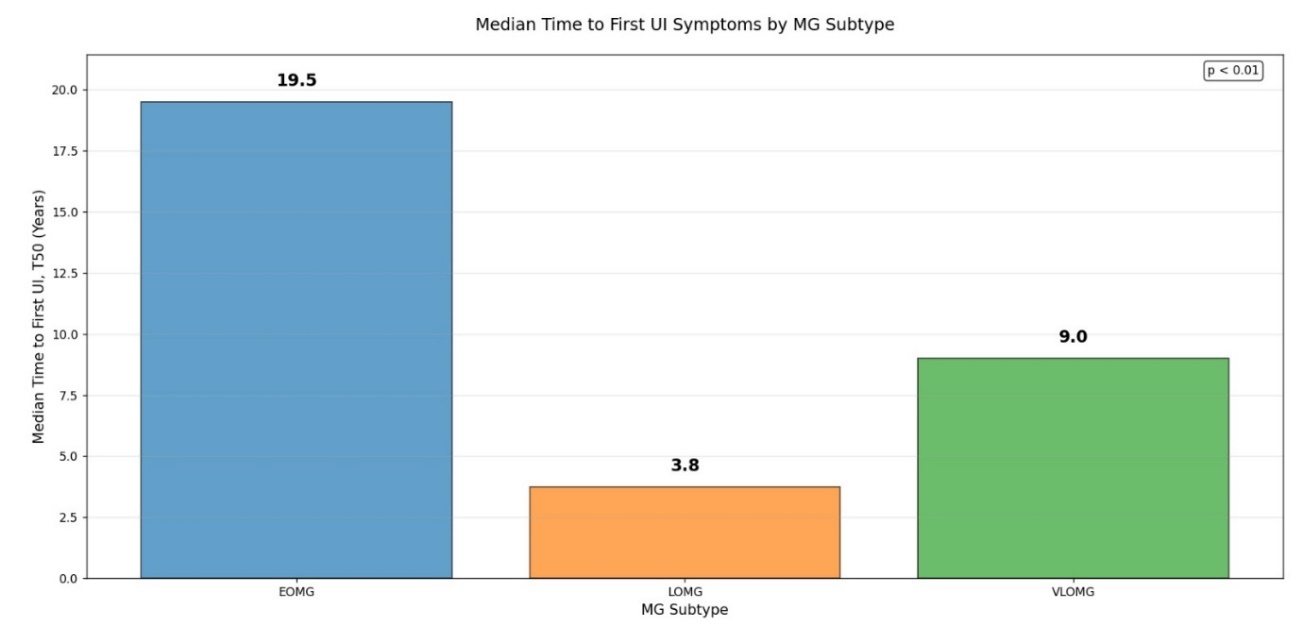


Supplementary figure 2a: LOMG patients reached UI earlier (3.8 years) than VLOMG (9.0) and EOMG (19.5) (p < 0.01).

**Abbreviations:** EOMG, early-onset MG; LOMG, late-onset MG; VLOMG, very late-onset MG; UI, urinary incontinence. Abbreviations: EOMG, early-onset MG; LOMG, late-onset MG; VLOMG, very late-onset MG; UI, urinary incontinence; T50, time at which 50% of patients developed UI.

**Supplementary figure 2b: Median Time (T50) From Onset of MG to First Urinary Incontinence (UI) Symptoms Stratified by Disease Severity**


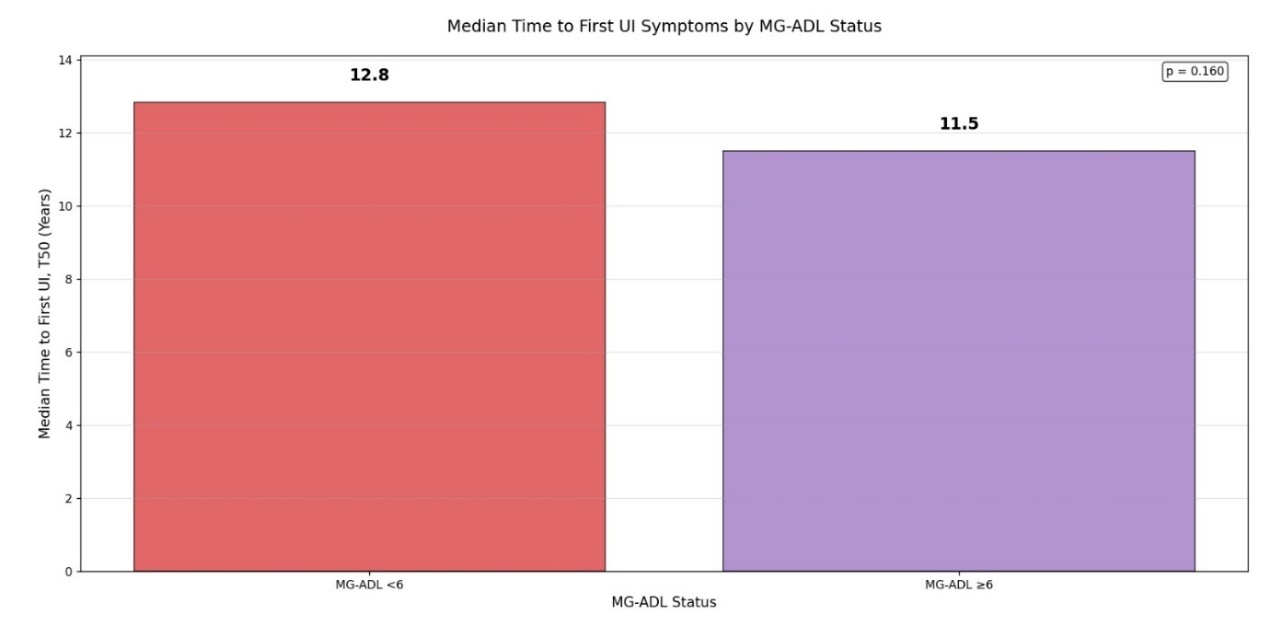


Supplementary figure 2b**:** patients with MG-ADL ≥6 developed UI earlier than those with MG-ADL <6 (11.5 vs. 12.8 years; p = 0.160).

**Abbreviations:** MG-ADL, Myasthenia Gravis Activities of Daily Living; UI, urinary incontinence; T50, time at which 50% of patients developed UI.
